# Supplementary material for: Divergent effect of fluoxetine on the response to physical or chemical stressors in zebrafish
Source: PeerJ. 2017 May 9;5:e3330. doi: 10.7717/peerj.3330 (PMC5426348; doi:10.7717/peerj.3330)
Supplement: Supplemental Information 1 [file peerj-05-3330-s001.pdf]

Raw data Experiment 1, chemical stressors

Control			Fluoxetine exposed		
**Control**	**Alarm substance**	**Blood**	**Control**	**Alarm substance**	**Blood**
2.5	0.5	3.0	4.0	6.0	5.0
1.0	4.0	2.0	4.5	5.5	3.0
2.0	9.0	3.0	0.25	7.5	4.0
1.5	5.0	7.5	2.0	4.0	7.0
1.0	4.0	5.5	3.0	0.25	2.0
1.25	7.0	5.0	1.5	5.5	1.0
	3.0	8.0		1.5	9.0
	3.0	3.0		2.0	4.0
	4.5			5.0	3.0
	5.0			3.0	7.0
				2.0	2.0
				3.0	

Statistics Experiment 1, chemical stressors

Two-way ANOVA	Ordinary				
Alpha	0,05				
Source of Variation	% of total variation	P value	P value summary	Significant?	
Interaction	2,324	0,4994	ns	No	
Row Factor	18,55	0,0064	**	Yes	
Column Factor	0,02834	0,8963	ns	No	
ANOVA table	SS	DF	MS	F (DFn, DFd)	P value

Interaction	6,261	2	3,130	F (2, 48) = 0,7045	P = 0,4994
Row Factor	49,96	2	24,98	F (2, 48) = 5,623	P = 0,0064
Column Factor	0,07633	1	0,07633	F (1, 48) = 0,01718	P = 0,8963
Residual	213,3	48	4,443		

Compare cell means regardless of rows and columns								
Number of families	1							
Number of comparisons per family	5							
Alpha	0,05							
Dunnett's multiple comparisons test	Mean Diff,	95% CI of diff,	Significant?	Summary				
Control:Control vs. Control:FLU	-1,000	-4,165 to 2,165	No	ns				
Control:Control vs. Alarm substance:Control	-2,958	-5,789 to -0,1272	Yes	*				
Control:Control vs. Alarm substance:FLU	-2,229	-4,970 to 0,5120	No	ns				
Control:Control vs. Blood:Control	-3,236	-6,126 to -0,3466	Yes	*				
Control:Control vs. Blood:FLU	-2,731	-5,513 to 0,05136	No	ns				
Test details	Mean 1	Mean 2	Mean Diff,	SE of diff,	N1	N2	q	DF
Control:Control vs. Control:FLU	1,542	2,542	-1,000	1,217	6	6	0,8217	48
Control:Control vs. Alarm substance:Control	1,542	4,500	-2,958	1,089	6	10	2,718	48
Control:Control vs. Alarm substance:FLU	1,542	3,771	-2,229	1,054	6	12	2,115	48
Control:Control vs. Blood:Control	1,542	4,778	-3,236	1,111	6	9	2,913	48
Control:Control vs. Blood:FLU	1,542	4,273	-2,731	1,070	6	11	2,553	48

Raw data Experiment 2, physical stressors

Control			Fluoxetine exposed		
Control	Spatial restriction	Persecution	Control	Spatial restriction	Persecution
2.5	3.5	33.0	4.0	8.5	10.0
1.0	5.0	28.0	4.5	1.0	8.0
2.0	7.0	20.0	0.25	2.0	0.5
1.5	5.0	20.0	2.0	5.0	1.0
1.0	24.5	13.0	3.0	2.5	7.5
1.25	8.5	16.0	1.5	3.5	8.5
	2.0	14.5		4.0	
	8.0	16.0		3.5	
	29.0	15.5		2.0	
	3.0			8.0	
	11.5			5.0	
	5.0			4.0	

Statistics Experiment 2, Physical stressors

Two-way ANOVA	Ordinary				
Alpha	0,05				
Source of Variation	% of total variation	P value	P value summary	Significant?	
Interaction	11,28	0,0046	**	Yes	
Row Factor	23,99	< 0,0001	****	Yes	
Column Factor	12,89	0,0005	***	Yes	
ANOVA table	SS	DF	MS	F (DFn, DFd)	P value
Interaction	360,1	2	180,1	F (2, 45) = 6,080	P = 0,0046
Row Factor	765,7	2	382,9	F (2, 45) = 12,93	P < 0,0001

Column Factor	411,4	1	411,4	F (1, 45) = 13,89	P = 0,0005
Residual	1333	45	29,61		
Number of missing values	21				

Compare cell means regardless of rows and columns								
Number of families	1							
Number of comparisons per family	5							
Alpha	0,05							
Dunnett's multiple comparisons test	Mean Diff,	95% CI of diff,	Significant?	Summary				
Control:Control vs. Control:FLU	-1,000	-9,191 to 7,191	No	ns				
Control:Control vs. Spatial restriction:Control	-7,792	-14,89 to -0,6981	Yes	*				
Control:Control vs. Spatial restriction:FLU	-2,542	-9,635 to 4,552	No	ns				
Control:Control vs. Persecution:Control	-18,01	-25,49 to -10,54	Yes	****				
Control:Control vs. Persecution:FLU	-4,375	-12,57 to 3,816	No	ns				
Test details	Mean 1	Mean 2	Mean Diff,	SE of diff,	N1	N2	q	DF
Control:Control vs. Control:FLU	1,542	2,542	-1,000	3,142	6	6	0,3183	45
Control:Control vs. Spatial restriction:Control	1,542	9,333	-7,792	2,721	6	12	2,864	45
Control:Control vs. Spatial restriction:FLU	1,542	4,083	-2,542	2,721	6	12	0,9341	45
Control:Control vs. Persecution:Control	1,542	19,56	-18,01	2,868	6	9	6,281	45
Control:Control vs. Persecution:FLU	1,542	5,917	-4,375	3,142	6	6	1,392	45
